# Early socialization and environmental enrichment of lactating piglets affects the caecal microbiota and metabolomic response after weaning

**DOI:** 10.1038/s41598-021-85460-7

**Published:** 2021-03-17

**Authors:** M. Saladrigas-García, M. D’Angelo, H. L. Ko, S. Traserra, P. Nolis, Y. Ramayo-Caldas, J. M. Folch, P. Vergara, P. Llonch, J. F. Pérez, S. M. Martín-Orúe

**Affiliations:** 1 Animal Nutrition and Welfare Service, Department of Animal and Food Science, Universitat Autònoma de Barcelona (UAB), 08193 Bellaterra, Spain; 2 Department of Cell Biology, Physiology and Immunology, Universitat Autònoma de Barcelona (UAB), 08193 Bellaterra, Spain; 3 Nuclear Magnetic Resonance (SeRMN), Universitat Autònoma de Barcelona (UAB), 08193 Bellaterra, Spain; 4 Animal Breeding and Genetics Program, Institute for Research and Technology in Food and Agriculture (IRTA), Torre Marimon, 08140 Caldes de Montbui, Spain; 5 Plant and Animal Genomics, Centre for Research in Agricultural Genomics (CRAG), CSIC-IRTA-UAB-UB Consortium, 08193 Bellaterra, Spain; 6 Department of Animal and Food Science, Universitat Autònoma de Barcelona (UAB), 08193 Bellaterra, Spain

**Keywords:** Gastrointestinal system, Animal physiology, Gene expression, Microbial communities, Metabolomics, High-throughput screening

## Abstract

The aim of this study was to determine the possible impact of early socialization and an enriched neonatal environment to improve adaptation of piglets to weaning. We hypothesized that changes in the microbiota colonization process and in their metabolic response and intestinal functionality could help the animals face weaning stress. A total of 48 sows and their litters were allotted into a control (CTR) or an enriched treatment (ENR), in which piglets from two adjacent pens were combined and enriched with toys. The pattern of caecal microbial colonization, the jejunal gene expression, the serum metabolome and the intestinal physiology of the piglets were assessed before (-2 d) and after weaning (+ 3d). A differential ordination of caecal microbiota was observed after weaning. Serum metabolome suggested a reduced energetic metabolism in ENR animals, as evidenced by shifts in triglycerides and fatty acids, VLDL/LDL and creatine regions. The TLR2 gene showed to be downregulated in the jejunum of ENR pigs after weaning. The integration of gene expression, metabolome and microbiota datasets confirmed that differences between barren and enriched neonatal environments were evident only after weaning. Our results suggest that improvements in adaptation to weaning could be mediated by a better response to the post-weaning stress.

## Introduction

In intensive pig farming, the process of weaning is a multifactorial stressor in piglet’s life affected by physiological, social, environmental and nutritional challenges. In the current productive systems, piglets are housed with their mothers in farrowing pens, separated from other sows and their progenies. After weaning, usually, at around 28 days of life, suckling pigs are moved prematurely from their mothers and mixed with new pen mates with whom they need to establish new hierarchies^1^. Moreover, piglets experience an abrupt change to a solid diet and are suddenly exposed to a different microbiological environment with a digestive and immune system still immature. In this scenario, weaning is frequently associated to alterations in intestinal function^2,3^. Dysbiosis, alteration of the intestinal barrier function, and diarrhoea are common due to the overgrowth of opportunistic pathogens such as *E. coli*^3^.

To improve the adaptation of piglets to weaning, alternative neonatal environments during the lactation period have been proposed. Among them, allowing sows and piglets from different litters to interact from the first day, has been proposed as a novel mean to facilitate the establishment of ubiquitous intestinal microbiota and reduce social stress after weaning^4–6^. In this regard, the existence of a relationship between the housing system and the microbiota of the sows has been demonstrated^7^. Keeping the sows and their litters individualized during the suckling period, might limit the microbiota exchange between adult sows and lead to a poorer microbial exposure for their piglets. This is particularly relevant considering that the intestinal microbiota of new-born animals has been demonstrated to play a fundamental role in the development of intestinal function and the innate immune system^8^. In humans, the reduced microbial exposure during early childhood has been associated with the appearance of immune deficiencies and health conditions^9^.

An enriched environment during the early life of piglets is known to positively influence behavioural development and stress adaptation later in life^10^, by providing piglets with the appropriate social skills and stress coping capabilities^11^. Moreover, favouring social interaction between litters during lactation can improve the social adaptation of the piglet at the time of weaning^12–14^, with a clear decrease in agonistic behaviour between piglets^4,5,15^.The combination of both physical and social enrichment has been reported to have a substantial impact on piglets’ socio-cognitive development^15^, improving their ability to cope with routine stressors. However, the underlying mechanisms that explain this reduction of stress response remain unknown. It was hypothesized that combining early socialization and environmental enrichment could improve the early intestinal colonization of suckling piglets and also their adaptation to the stress of weaning contributing all together to reduce its negative impact on intestinal health. Thus, the aim of the present study was to determine the combined effects of early socialization and neonatal enriched environment during lactation on the pattern of caecal microbial colonization, the jejunal gene expression, the serum metabolome and the intestinal physiology of the piglets before and after weaning and investigate the potential association with the adaptive response at weaning.

## Results

This work was part of a larger behavioural study that has been published^16^ and is recommended for complementary information. That study included behavioural observations, registers of skin and ear biting lesions as indicators of aggression and salivary stress biomarkers. In that work it was shown a lasting positive effect of the ENR treatment on piglets’ behaviour with an increase in object exploration before weaning and a mitigated weaning stress with reduced aggression from post-weaning until slaughter.

From the forty-eight sows initially included in the study, one control sow and its litter were discarded due to lameness prior to parturition. The average litter size was 14.1 ± 0.1 piglets for both CTR and ENR groups.

The impact of the treatments on performance of these animals has been also previously reported^17^. It was found a higher average daily gain (ADG) in ENR piglets during the first 5 days after weaning (23-27d; *P* = 0.030) compared to CTR piglets. Moreover, a trend for an increased ADG was also observed in ENR piglets during the nursery to fattening period (d69-79; *P* = 0.060). When analysing ADG from birth until market weight (90 kg), no differences were found between CTR or ENR piglets although the slaughter age for ENR piglets was lower than for CTR piglets (194.4 ± 1.0 vs. 197.7 ± 1.3 days (*P* = 0.080)) suggesting a potentially improved long-term growth performance due to enrichment.

### Caecal microbiota (16S rRNA gene sequencing)

#### Microbiota structure and biodiversity

On average, 78,562 ± 24,539 sequences per sample with an average length of 460 bp were obtained from 28 caecal content samples, with no differences between treatments or sampling day (*P* = 0.742 and 0.424, respectively), despite variability ranging from 40,061 to 132,201 sequences per sample. The rarefaction curves reached the plateau phase, proving that almost all bacterial species were detected. The sequences were assigned to 976 Operational Taxonomic Units (OTU) based on a 97% sequence similarity. The number of OTU that were common in groups as well as within the groups were evidenced using the Venn diagram, which showed there were 11 and 37 unique OTU in suckling piglets (-2 d) and weaned piglets (+ 3 d), respectively.

The indexes of Chao1, observed species, Shannon and Simpson were calculated to estimate alpha diversity. No significant differences were observed between control or enriched piglets (*P* > 0.1), either when measured for the whole study period or separated by sampling day. However, differences were found as expected related to the weaning process between suckling and weaned piglets, with a significant increase in richness after weaning (*P* = 0.015, *P* = 0.017, *P* = 0.013, *P* = 0.080; for Chao1, observed species, Shannon and Simpson indices, respectively). Regarding beta diversity, no difference was found related to differential management (*P* = 0.538) and a tendency was detected between nursing and weaned piglets (*P* = 0.062) for a higher diversity as animals grow.

**Figure 1:**
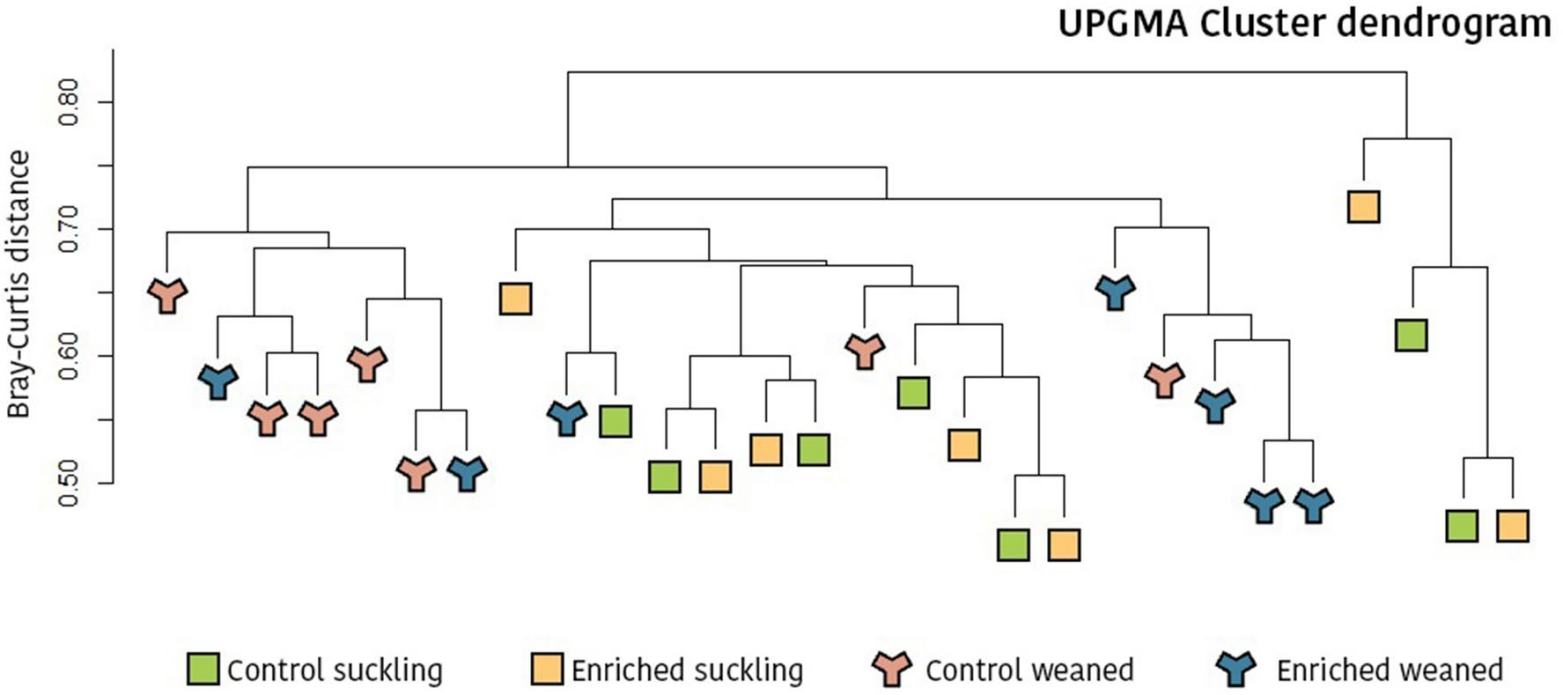
Hierarchical grouping dendrogram by UPGMA (average method) based on Bray–Curtis distances and relative OTU counts. A clear clustering is observed between suckling and weaned piglets. Likewise, enriched weaned piglets assimilate more to suckling animals than to the other control weaned piglets. Figure created by using open-source software R v3.5.3. (https://www.r-project.org/foundation/).

The microbial structure of the caecal content and differences in overall beta-diversity were calculated using Anosim, Adonis and Envfit tests, all of them based on Bray–Curtis distance. For the whole study, no significant differences were detected due to neonatal conditions (CON vs. ENR) (*P* = 0.387, *P* = 0.523 and *P* = 0.445, for Envfit, Anosim and Adonis tests, respectively). However, when analysing differences due to the experimental treatments by sampling day, although no differences were found during the suckling period, a statistical trend for an increased beta-diversity in the control piglets was found after weaning (*P* = 0.033, *P* = 0.053 and *P* = 0.058, for Envfit, Anosim and Adonis tests, respectively). As expected, weaning was associated to a change in the microbiota structure and significant differences between suckling and weaned piglets were found (*P* = 0.0001, *P* = 0.001 and *P* = 0.0001, for Envfit, Anosim and Adonis tests, respectively). At last, a cluster dendrogram was constructed using the UPGMA method (Fig. 1). As a result, a clear clustering is observed between suckling and weaned piglets. However, it is also interesting to note that enriched weaned piglets assimilated more to suckling animals than to the other control weaned piglets.

#### Taxonomy of caecal microbiota

*Firmicutes* and *Bacteroidetes* constituted the two predominant phyla in the caecal microbiota of both suckling and weaned piglets, contributing with 44.6% *Firmicutes* and 38.2% *Bacteroidetes* of the relative abundance. *Proteobacteria* (5.75 and 9.76%, for CTR and ENR, respectively), *Spirochaetes* (2.90 and 4.08%) and *Fusobacteria* (3.42 and 2.29%) were considered as predominant phyla as well. Other phyla were represented in less than 1% of relative abundance. Phylum relative counts and their respective *P*-values for suckling and weaned piglets can be found in Supplementary Table S1.

No significant differences were found in any phyla related to the neonatal environment, neither in the lactation period nor after weaning. At genus level, 69 genera were detected, among which there were 16 genera with a relative abundance higher than 1%, although only 11 of them were above this value in both groups. *Prevotella* was the most predominant genus both in suckling and weaned piglets, with an average relative abundance of 15.4% in the control group and 11.9% in the enriched group. Genus relative counts and the differences observed before and after weaning are shown in Supplementary Table S2. *Fusobacterium* and *Bacteroides* showed a decrease in the percentage of total sequences observed between suckling and weaned piglets. Similarly, whereas *Lactobacillus* and *Megasphaera* represented around 2% in suckling pigs, they did not reach 1% after weaning. Again, concerning the neonatal environment, although some minor differences were seen, they were not statistically relevant in neither suckling nor weaned piglets.

### Jejunal gene expression

Jejunum samples from the piglets were collected to analyse the expression of genes related to intestinal health and functionality by using the Open-Array technology. Results are shown in Table 1.

**Table 1:** Mean DCrt results obtained for the 51 genes that could be quantitatively determined, both during lactation and after weaning for CTR and ENR piglets. Details for the different genes can be found in Supplementary Table S4. (BF: Barrier function related genes/EH: Enzymes/Hormones related genes/IR: Immune system related genes/NT: Nutrient Transport related genes/ST: Stress related gene).

Function	Gene	LACT				WEAN			
		CTR	ENR	SEM	*P*-value	CTR	ENR	SEM	*P*-value
BF	*TFF3*	3.69	3.74	0.151	0.9254	3.17	3.21	0.197	0.9799
BF	*OCLN*	7.89	7.67	0.125	0.7494	6.97	7.13	0.075	0.8255
BF	*ZO1*	4.28	4.11	0.132	0.8836	4.06	3.83	0.090	0.7439
BF	*CLDN1*	17.51	16.07	0.550	0.7494	16.48	15.56	0.397	0.7466
BF	*CLDN4*	15.63	15.28	0.188	0.7494	14.64	14.83	0.204	0.8644
BF	*CLDN15*	9.00	8.58	0.234	0.7494	9.49	9.36	0.112	0.8255
BF	*MUC2*	5.37	5.61	0.211	0.8836	4.87	4.64	0.199	0.8255
BF	*MUC13*	2.43	2.30	0.294	0.9194	1.22	1.07	0.092	0.8255
EH	*SI*	3.07	2.74	0.323	0.8836	1.40	1.54	0.199	0.9888
EH	*DAO1*	2.95	2.74	0.373	0.9491	2.37	2.54	0.138	0.8255
EH	*HNMT*	5.49	5.16	0.157	0.9062	4.63	4.64	0.099	0.9799
EH	*ANPEP*	1.43	0.48	0.378	0.7788	0.37	0.33	0.120	0.9799
EH	*IDO1*	9.19	8.65	0.443	0.9062	8.21	7.35	0.318	0.7439
EH	*GCG*	4.95	4.17	0.220	0.6220	4.19	4.01	0.122	0.8255
EH	*CCK*	7.36	7.36	0.165	0.9925	9.15	9.77	0.303	0.8255
EH	*IGF1R*	6.39	6.15	0.171	0.8836	7.93	7.11	0.199	0.7439
EH	*PYY*	7.16	6.61	0.233	0.7494	6.83	7.04	0.194	0.8255
EH	*GPX2*	4.88	5.21	0.327	0.8836	5.65	4.45	0.395	0.7439
EH	*SOD2.m*	4.62	4.63	0.170	0.9925	5.00	4.49	0.133	0.7439
EH	*ALPI*	2.28	1.17	0.545	0.9194	0.54	1.23	0.196	0.7439
IR	*TLR2*	13.57	13.15	0.234	0.7494	13.94	11.69	0.391	0.0315
IR	*TLR4*	7.63	7.55	0.179	0.9254	8.07	7.22	0.291	0.7439
IR	*IL1B*	10.79	9.57	0.354	0.6220	9.61	9.00	0.342	0.8255
IR	*IL6*	13.62	12.70	0.373	0.7494	13.20	12.67	0.270	0.8255
IR	*IL10*	10.24	9.91	0.171	0.7494	9.55	9.58	0.173	0.9799
IR	*IL17A*	17.86	17.43	0.469	0.8836	16.58	16.67	0.470	0.9799
IR	*IL22*	12.57	12.10	0.583	0.8836	11.84	11.91	0.324	0.9799
IR	*IFN-γ*	9.86	9.05	0.448	0.7494	9.13	8.85	0.261	0.8255
IR	*TNF-α*	9.62	8.97	0.231	0.7494	9.08	8.56	0.186	0.7439
IR	*TGF-β1*	5.29	5.33	0.101	0.9254	5.22	5.01	0.148	0.8255
IR	*CCL20*	5.88	4.77	0.561	0.7494	4.64	4.79	0.392	0.9799
IR	*CXCL2*	10.23	9.10	0.373	0.7494	10.15	9.65	0.309	0.8255
IR	*IFNGR1*	4.78	4.58	0.214	0.8813	3.29	3.36	0.094	0.9115
IR	*HSP27*	3.35	2.83	0.228	0.7494	2.97	3.35	0.128	0.7439
IR	*HSP70*	3.51	3.24	0.186	0.8698	3.24	3.24	0.075	0.9964
IR	*REG3G*	6.38	6.58	0.502	0.9254	7.02	3.74	0.970	0.7439
IR	*PPARGC1α*	7.28	7.28	0.147	0.9925	7.90	8.09	0.159	0.8255
IR	*FAXDC2*	6.23	4.52	0.490	0.6825	4.05	4.76	0.298	0.7164
IR	*GBP1*	3.19	2.59	0.329	0.8797	2.77	2.63	0.131	0.8255
IR	*IL8*	4.67	4.39	0.245	0.8836	4.78	4.53	0.200	0.8255
NT	*SLC5A1*	2.38	1.97	0.530	0.9491	1.35	1.46	0.252	0.8225
NT	*SLC16A1*	6.44	6.78	0.196	0.9062	7.86	7.65	0.166	0.8255
NT	*SLC7A8*	8.03	5.84	0.621	0.6220	7.73	7.18	0.432	0.8255
NT	*SLC15A1*	5.18	4.00	0.463	0.7788	3.14	3.80	0.264	0.7907
NT	*SLC13A1*	7.55	5.07	0.576	0.6825	4.15	4.62	0.181	0.7798
NT	*SLC11A2*	6.63	6.58	0.092	0.9254	6.99	6.84	0.117	0.8255
NT	*SLC30A1*	5.07	4.48	0.171	0.7326	3.59	3.77	0.136	0.8255
NT	*SLC39A4*	4.54	4.70	0.202	0.8836	5.91	6.29	0.152	0.7439
ST	*CRHR1*	15.00	15.68	0.362	0.7494	15.20	15.14	0.333	0.9799
ST	*NR3C1-Grα*	6.59	6.11	0.123	0.7326	6.51	6.41	0.089	0.8255
ST	*HSD11B1*	8.56	8.98	0.184	0.8486	10.24	9.42	0.270	0.7439

No differences in expression were observed between experimental groups in any of the jejunal genes during lactation. However, the effect of differential neonatal environment of piglets was observed after weaning for TLR2 gene, that showed a higher expression in the control group (13.94 vs. 11.69, *P* = 0.0315).

### Metabolomic response

The representative proton nuclear magnetic resonance (^1^H-NMR) profiles of serum samples were obtained from the enriched and control groups both during lactation and after weaning (Supplementary Fig. S1), and an ampliation of one of them is shown in Supplementary Fig. S2. A number of endogenous metabolites were assigned from the ^1^H-NMR spectra, such as LDL/VLDL, leucine, valine, isoleucine, lactate, alanine, adipate, acetate, N-acetyl glycoproteins, O-acetyl glycoproteins, glutamine/glutamate, pyruvate, glutamate, creatine, choline, trimethylamine-N-oxide (TMAO), glucose, creatinine, tyrosine and phenylalanine based on comparing chemical shifts and multiplicities of peaks to public access databases like Human Metabolome Data Base (HMDB)^18^ and Biological Magnetic Resonance Data Bank (BMRB) and published studies^19–21^.

**Figure 2:**
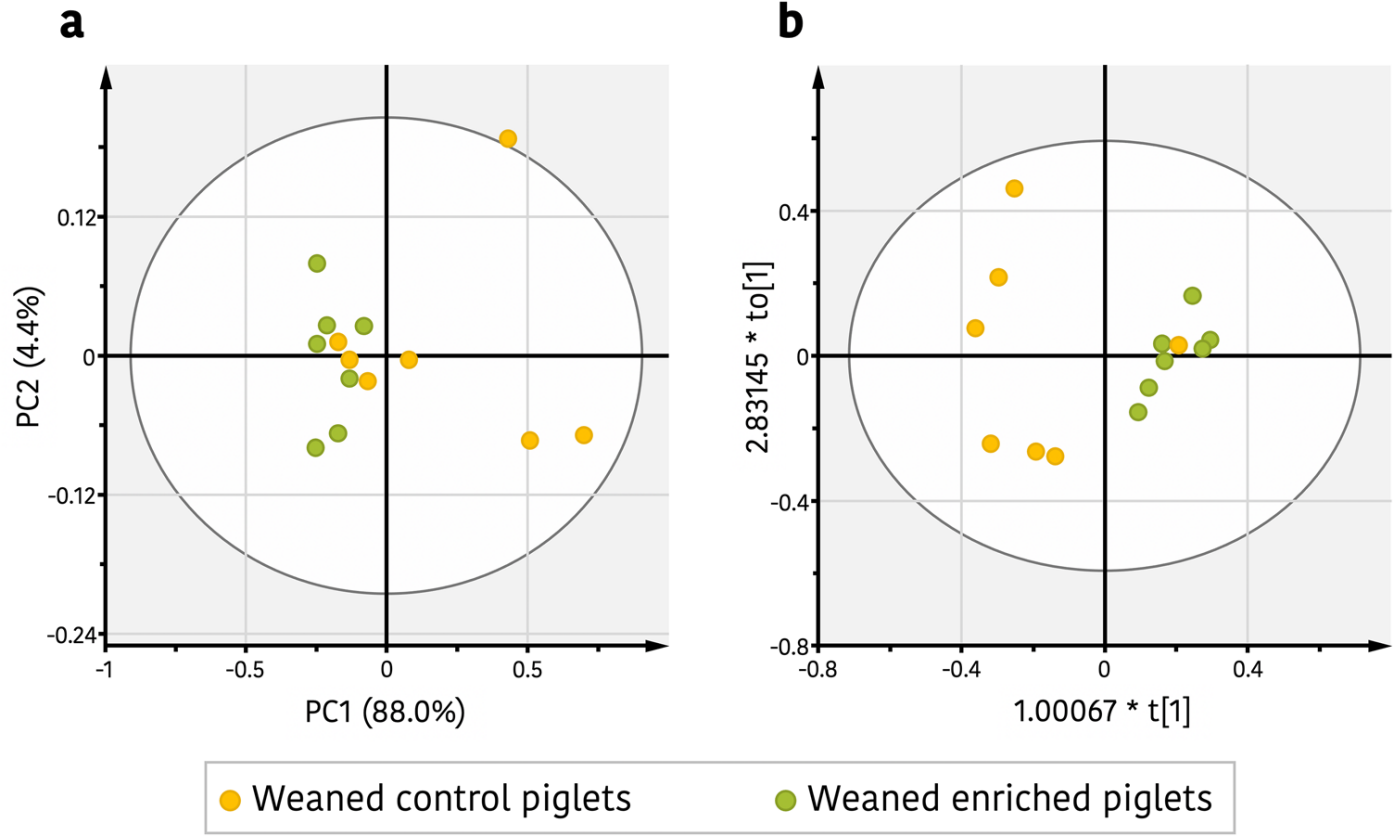
Effect of environmental and social enrichment on the serum metabolic profiles of piglets. (**a**) Principal components analysis (PCA) score plot of serum data set from weaned enriched (blue) and weaned control piglets (red). (**b**) Orthogonal partial least squares discrimination analysis (OPLS-DA) score plot between weaned enriched piglets (blue) and control group (red). Figure created by using open-source software R v3.5.3. (https://www.r-project.org/foundation/).

With the purpose of investigated potential differences in the ^1^H-NMR metabolites profiles between enriched and control piglets during lactation and after weaning, a nontargeted metabolomic approach was made. Previously, in order to reduce the number of variables, a filtering of ^1^H-NMR bucket table was done by significant differences on Student’s *t*-test between the integrated buck regions of enriched and control piglets (Supplementary Table S3). During lactation, principal components analysis (PCA) was made to evaluate the global metabolic profile of the two groups but did not show a clear clustering. Additionally, an orthogonal projection to latent structures discriminant analysis (OPLS-DA) model was constructed but the model did not show neither an acceptable predictive ability (Supplementary Table S3). However, when the same multivariate analysis was made after weaning, a trend of separation between enriched weaned piglets and control weaned piglets along PC1 could be observed indicating that both groups were metabolically differenced. This can be seen in Fig. 2a, which shows a biplot of PCA [R^2^x_(cum)_ = 0.95, Q^2^_(cum)_ = 0.83] from the reduced data where each spot represents the metabolic serum profile for each sample. A supervised OPLS-DA model was constructed to identify any subtle change in serum metabolites due to enrichment, a model with accepted fitness R^2^ and predictive ability Q^2^ parameters was obtained [R^2^x_(cum)_ = 0.91, R^2^y_(cum)_ = 0.68, Q^2^_(cum)_ = 0.53], that produced good separation into the two clusters along PC1 (Fig. 2b). Moreover, both the cross-model validation (Supplementary Fig. S3b) and the 100 times permutation test (Supplementary Fig. S3**c**) indicated that the constructed OPLS-DA model was positive and valid and confirmed the distinction among enriched and control weaned piglets. Furthermore, the area under the curve (AUC) for the receiver operating characteristic (ROC) plot (Supplementary Fig. S4) with a value of 0.92 indicated a robust discrimination power (high sensitivity and specificity) for the OPLS-DA classifier model. An S-plot was constructed to identify the ^1^H-NMR regions that contributed significantly to the differentiation of enriched and control weaned piglets (Supplementary Fig. S5) and the detected regions were screened according to their corresponding variable importance in the projection (VIP) values of the OPLS-DA model. The metabolites corresponding to each one of these shifts were identified as explained previously and were triglycerides and fatty acids, VLDL, unsaturated lipids, LDL and creatine. All metabolites were significantly higher in the control piglets when compared to the enriched piglets (Table 2).

**Table 2:** Key metabolites that differentiate serum of enriched piglets (ENR) from control (CTR) piglets at post-weaning period. *P*-values were derived from Student’s *t*-test. Variable importance in the projection (VIP) value was derived from OPLS-DA with a threshold of 0.75.

^1^H Chemical shift ppm (Central bucket point)	Metabolite	Moieties	KEEG IDs	ENR versus CTR		
				Fold Change CTR/ENR	*P*-value	VIP
1.30	Lipids^a^	$-({\mathrm{CH}}_{2}{)}_{n}-$	NA	2.7	0.021	2.09
1.26	Lipids^a^	$-({\mathrm{CH}}_{2}{)}_{n}-$	NA	2.9	0.024	1.97
0.90	VLDL	${\mathrm{CH}}_{3}^{*}{\mathrm{CH}}_{2}{\mathrm{CH}}_{2}\mathrm{C}=$	NA	2.6	0.014	1.25
5.30	Unsaturated lipids	$-\mathrm{CH}=\mathrm{CH}-$	NA	3.6	0.027	1.17
2.02	Unsaturated lipids	$-{\mathrm{CH}}_{2}^{*}-\mathrm{CH}=\mathrm{CH}-$	NA	1.7	0.039	1.12
0.86	LDL	${\mathrm{CH}}_{3}^{*}({\mathrm{CH}}_{2}{)}_{n}-$	NA	1.9	0.018	1.09
3.94	Creatine	$-{\mathrm{CH}}_{2}-$	C00300	1.5	0.006	0.82
1.58	Lipids^a^	$-{\mathrm{CH}}_{2}^{*}{\mathrm{CH}}_{2}\mathrm{CO}-$	C06104	4.7	0.033	0.77

^a^Triglycerides and fatty acids.

VLDL: very low-density lipoprotein; LDL: low density lipoprotein.

### Integration of the omics technologies

Gene expression, caecal microbiota and metabolomics were integrated by using the open-source software R v3.6.1 and the LinkHD package. As a result, samples were stratified into clusters. The relationship between clusters and the variables responsible for the attained structure were obtained.

**Figure 3:**
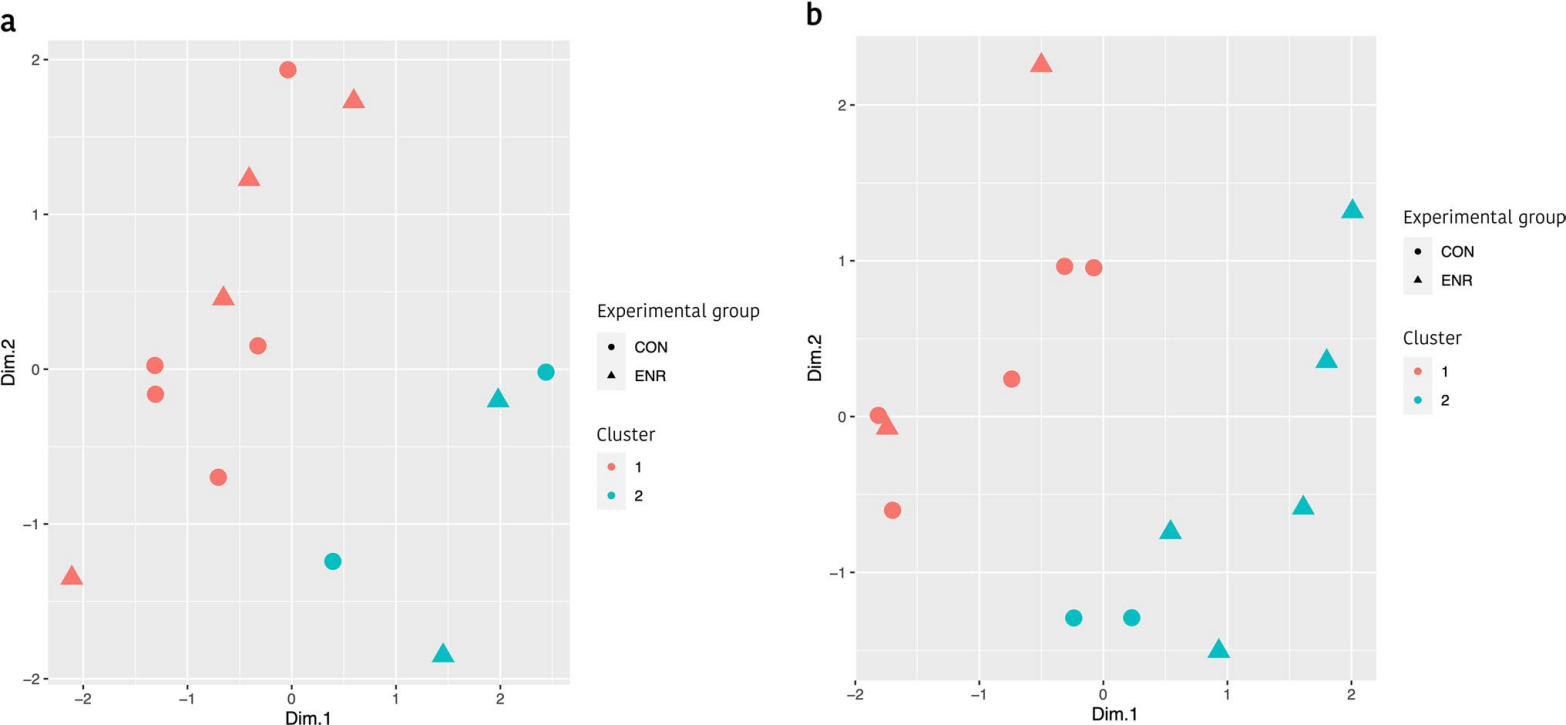
Scatterplot of cluster stratification according to LinkHD blind analysis. **a** The clustering of the samples during lactation, whereas **b** shows the clustering of the samples after weaning. A similar cluster distribution was observed with the hierarchical grouping dendrogram by using the UPGMA (average method) based on Bray–Curtis distances and relative OTU counts (Fig. 1). Figures created by using open-source software R v3.5.3. (https://www.r-project.org/foundation/).

During lactation, clusters were not related to the experimental treatments (Fig. 3a), while after weaning, the samples were gathered in two differentiated clusters (Fig. 3b). After implementing variable selection based on regression biplot, those variables that were most associated with the common structure of the data (i.e.: a compromise that maximize the relationship between the different omics layers) were those related to the microbiota of the caecal content. Although no differential abundance was observed at taxonomic level after weaning, LinkHD separated the samples into two differentiated clusters, that were characterized mainly by a greater abundance of *Lactobacillaceae*, *Fusobacteriaceae*, *Alcaligenaceae*, *Bacteroidaceae*, and *Campylobacteraceae*, and a less abundance of *Erysipelotrichaceae* and *Clostridiaceae* in the enriched piglets after weaning compared with the control group.

Regarding the impact of weaning itself, Fig. 1 shows three clusters that clearly separated piglets in lactation or after weaning. Again, this differential clustering was mostly explained by the changes on the piglet gut microbiota. Confirming the previous approach, the disparity between suckling and weaned piglets was found to be due to reductions in *Fusobacteriaceae*, *Bacteroidaceae*, *Enterobacteriaceae*, and *Lactobacillaceae*; and increases in *Lachnospiraceae* and *Erysipelotrichaceae* after weaning.

### Functionality of the large intestine

**Figure 4:**
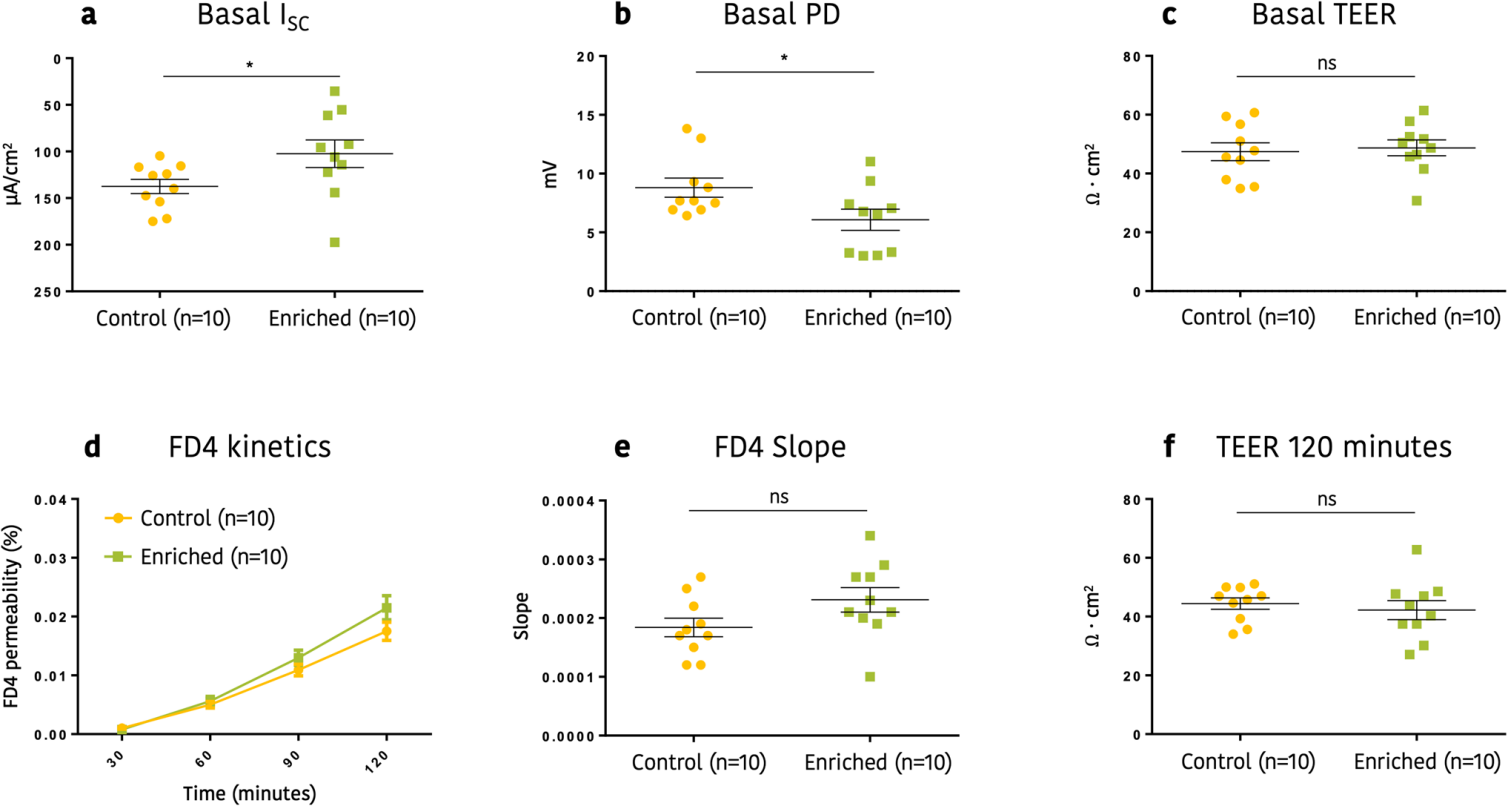
Effect of early socialization and environmental enrichment on transepithelial ion transport paracellular permeability to fluorescent tracers at day 3 post weaning. (**a**) Basal colonic I_sc_, (**b**) Basal colonic PD, (**c**) Basal colonic TEER, (**d**) FD4 flux, (**e**) FD4 slope and (**f**) TEER at 120 min.

Different assays with Ussing chambers and colon mucosa were done to evaluate the possible impact of the experimental treatments in intestinal physiology. Accordingly, Fig. 4 shows the assessment of the electrolyte transport across the intestinal epithelium as well as of the barrier integrity in both experimental groups.

At day 3 post weaning (+ 3 d), an increase in basal colonic short-circuit current (I_sc_) and potential difference (PD) was observed in the CTR group compared to the ENR one (Fig. 4a and b), suggesting a higher level of ion transport across the colonic tissue of control animals (*P* = 0.029 and *P* = 0.050, respectively). Basal colonic TEER did not shown however differences between groups (control group: 47.4 ± 3.0 Ω·cm^2^, enriched group: 48.7 ± 2.7 Ω·cm^2^) (Fig. 4c).

Regarding changes in the paracellular permeability to fluorescent tracers, mucosal to basolateral passage of FD4 across the colon was measured every 30 min. Fluorescent tracer passage was time dependent in both groups (Fig. 4d) but differences between treatments observed after 120 min were not significant, being 0.018 ± 0.0016% in the control group and 0.022 ± 0.0020% in the enriched group (*P* = 0.220) (Fig. 4d). In the same line, both groups showed a tendency to a similar slope of the FD4 linear regression (Fig. 4e; *P* = 0.0900). In order to have another measurement of the colonic barrier integrity, TEER was also measured every 30 min, with almost negligible changes between experimental time and treatments. At the last time point, 120 min, TEER was around 43 Ω·cm^2^ in both groups (control group: 44.44 ± 1.925 Ω·cm^2^, 42.23 ± 3.229 Ω·cm^2^ enriched group) (Fig. 4f).

## Discussion

The early life is a critical period for the development of intestinal microbiota and immune system in pigs^22^. Differences in the way piglets are reared at the beginning of their lives is therefore expected to affect the gut microbial colonization and the intestinal immune development^23^. Moreover, differences in the way piglets are socially exposed and cognitively stimulated during their first days of life, could also determine differences in their abilities to cope with social and environmental challenges at weaning^16^. In this study, we assessed the potential benefits of a combined early socialization and an enriched environment during lactation on the pattern of caecal microbial colonization, the intestinal functionality and the metabolomic response of the piglets in order to improve their adaptive response to weaning stress.

In this study, the impact of socializing litters on the intestinal microbial colonization process during lactation appeared to be scarce and we were not able to find differences in the microbiota structure between groups along the suckling period. During this time *Bacteroidetes*, *Firmicutes* and *Proteobacteria* constituted the three predominant phyla in the caecal microbiota of suckling piglets, which is in accordance with previous studies^24–28^, followed by *Fusobacteria* that also has been described as one predominant phylum during lactation^26,27,29,30^. At genus level, although a high individual variability was observed, *Bacteroides* and *Lactobacillus* showed a higher relative abundance, in consonance with similar studies^26,31–33^, which can be correlated with a milk-oriented microbiome^32^. Other genera, such as *Fusobacteria* and *Megasphaera* were also abundant in suckling piglets, as stated by Chen et al^26^. However, no significant differences were found for particular taxonomic groups between experimental treatments during this period. According to our results, despite the ENR treatment demonstrated to have an impact on the behaviour of piglets, giving piglets the opportunity to socialize with other litters do not have a remarkable impact on the microbial colonization process. During this period, we neither found significant dissimilarities on gene expression nor the metabolic profiles. However, the piglets in the ENR group spent more time engaging in pen and object exploration and also showed an increased number of aggressions before combining litters^16^. These results would suggest that observed behavioural changes during lactation do not seem to have a remarkable impact on metabolomic or genomic response of the animals.

Bian et al.^34^ reported that the nursing mother and the breed do not influence gut microbiota as much as the introduction of solid feed and subsequent weaning, which dominated the succession of gut microbiota. Moreover, some studies have reported that the mothers do not represent the most important source of colonization during early life of piglets^7^. In fact, the composition of the microbiota after birth tended to be similar to microbes present on the slatted floor, sows’ milk and nipple surface, although this composition did not have a long stay during lactation^35^. Therefore, our results could not confirm our initial hypothesis about the possible impact of an early socialization in the gut colonization process but suggest that the changes observed in microbial community (*P* = 0.033, *P* = 0.053 and *P* = 0.058, for Envfit, Anosim and Adonis tests, respectively) after weaning are more likely due to the decrease of aggression and stress response registered after weaning in the ENR group^16^. In this regard the usual increase of lesions after weaning was more than 3 times greater in CTR compared to ENR pigs. On the other hand, the post-weaning increase of stress-related markers such as salivary cortisol and chromogranin A was only significant in CTR piglets^16^ evidencing the clear potential of this enrichment strategy to mitigate weaning stress.

A reduced stress could had led to changes in metabolic response. In this regard, the serum metabolome analysis of piglets by ^1^H-NMR showed changes that could be compatible with a decrease in the amounts of triglycerides, fatty acids, VLDL/LDL and creatine in the ENR pigs. Interestingly, these metabolites are directly related to lipid and energy metabolism. The increased concentration of creatine and VLDL suggests an increased energy demand in CTR piglets after weaning, as higher VLDL/LDL may be an adaptive response of the liver to provide energy to peripheral tissues^36^ and the increase in creatine concentration may suggest an extensive glycogenolysis and glycolysis^37^. Creatine plays a major role in energy metabolism by converting adenosine diphosphate (ADP) and phosphocreatine into adenosine triphosphate (ATP)^38^. Although there are very few studies in this field, Peeters et al.^39^ observed lower levels of creatine-kinase in pigs given a straw bedding when compared to control pigs. Straw-enriched pigs also showed a decreased pen interaction that could be thought to require a lower energy expenditure. These metabolic changes could be related to the reduced stress evidenced by the lower cortisol salivary levels and the fewer fights registered in the ENR pigs^16^. An improved metabolic response could be also due to a better adaptation to dry-food intake in the ENR group as suggest the higher ADG registered in the ENR piglets along the first 5 days post-weaning (*P* = 0.030). However, as stated by Mkwanazi et al.^40^, there is a large gap of research, especially according to the role of environmental enrichment and early socialization on changes in blood metabolites and further comprehension on this matter is needed.

Regarding the possible impact of early-socialization and environmental enrichment on the microbial colonization after weaning, the high throughput sequencing (HTS) results showed that, in general terms, the diversity and community structure of caecal microbiota were in consonance with the predominant taxa described previously for healthy piglets^24^. The species richness and diversity of caecal microbiota were increased in piglets during weaning transition as reported by other studies^26,29–31^. A higher diversity in the gut microbiota has been related to a more mature gut microbiota and is in agreement with the concept of functional redundancy, which supports that additional taxa add redundancy to specific functions, helping the ecosystem to preserve its resilience and stability after environmental stresses^41,42^. The succession of microbial colonization observed in both CTR and ENR piglets also fitted perfectly with the existing literature, and as reported by Bian et al.^34^ was caused majorly by the impact of weaning. The abrupt change to a solid cereal-based diet and the withdrawal of milk explain the decrease of genera like *Bacteroides* and *Lactobacillus* and the increase of butyrate-producing genera including *Roseburia*, *Ruminococcus*, and *Lachnospira*, among others, as reported by several other authors^26,31,43^. Altogether, the higher abundance of *Roseburia*, *Ruminococcus*, *Coprococcus*, *Dorea* and *Lachnospira* genera in weaned piglets show the microbial evolution of the piglets’ gut microbiota to cope with diets rich in complex carbohydrates.

Although no changes in the relative abundance of particular taxonomic groups after weaning related to the neonatal environment were identified, we observed changes in the global structure of caecal microbiota suggesting that early socialization of piglets, and an enriched neonatal environment during lactation, can influence the development of the intestinal microbiota even if we were not able to evidence changes along the suckling period. A similar outcome was obtained by D’Eath et al.^44^, who also studied the effect of early socialization of piglets between 10 and 30 days of age by removing the barriers between two adjacent pens. Their results in piglets also became especially evident after weaning but not during lactation. Therefore, the combined effects of early socialisation and environmental enrichment could exert their effects on piglet’s microbiota by improving their adaptability to stress and consequently, stress-related intestinal dysfunction.

To assess the impact of physical and social enrichment on intestinal functionality, gene expression analysis was performed. Fifty-six genes, related to gut health, were analysed from jejunum samples by using the Open-Array technology. As previously observed, no differences could be detected between CTR and ENR piglets during lactation, but only a down-regulation of the TLR2 gene in the ENR group after weaning. The TLR2 gene encodes the toll-like receptor 2 (TLR2) protein, a transmembrane receptor that plays a fundamental role in pathogen recognition and activation of innate immunity^45^. TLR2 have been shown to recognize conserved molecules derived from microorganisms known as pathogen-associated molecular patterns (PAMPs), activating the signalling pathways to modulate the host's inflammatory response. Many factors have been reported to trigger an upregulation of the TLR2 gene, as the presence of pathogenic bacteria such as *Salmonella* and ETEC, weaning or dietary probiotic administration, among others^46–49^. These weaning associated factors may disrupt the intestinal barrier, which enables toxins, bacteria, or feed-associated antigens to cross the epithelium^48^. Although the results of this study confirmed that the integrity of the intestinal barrier was not affected by the experimental treatments, since neither occludin expression nor FD4 permeability was altered, down-regulation of TLR2 expression in the ENR group and the significant reduction found ion transport across the colonic tissue (Ussing chambers) could suggest a reduction in pathogenic insults in this experimental group.

Ultimately, the integration of gene expression, metabolome and metagenome datasets with LinkHD program was not able to demonstrate any difference between experimental groups in the suckling period. However, after weaning, a differential response between CTR and ENR piglets was evidenced, as samples were distributed into two clusters mostly driven by the experimental treatment. LinkHD package was able to discriminate both clusters (CTR vs. ENR) based on differential abundances patterns in particular taxonomic groups. Particularly, greater abundances of *Fusobacteriaceae*, *Alcaligenaceae*, *Bacteroidaceae*, and *Campylobacteraceae* were pointed out by LinkHD in the cluster including most of enriched piglets and *Lactobacillaceae*, *Erysipelotrichaceae* and *Clostridiaceae* were found as remarkably lower in this cluster. In general terms, *Lactobacillaceae* and *Bacteroidaceae* families can be classified as favourable bacteria and genera belonging to *Fusobacteriaceae*, *Clostridiaceae* and *Campylobacteraceae* are commonly associated with intestinal diseases^50–53^. It is therefore difficult to extract conclusions from these results, as we cannot objectively associate these changes to a more or less beneficial microbiota. Moreover, to analyse these changes, we need to keep in mind that faecal samples were collected just 3 days after weaning when probably the microbial ecosystem was undergoing an intense evolution from a milk-based diet to a dry feed. From this point of view conventional microbial indicators for a more or less robust ecosystem should be regarded with precaution considering the complexity of the ecological interactions within the gut microbiota. Further research would be needed to see whether these changes can be associated to a differential disease sensitivity.

## Conclusion

Rearing suckling piglets in an enriched environment and an early piglet socialization program do not seem to have a relevant impact on the microbial colonization pattern during the lactation period and neither on the metabolomic response of the animals. However, this differential neonatal environment results in a divergent response after weaning with differences in the microbial structure and a reduced jejunal expression of the TLR2 gene in ENR piglets. Changes detected in metabolites like triglycerides, fatty acids, VLDL/LDL or creatine also suggest an impact on energy metabolism consistent with the previously reported reductions of aggressions in these animals. These results suggest that creating a physically and socially enriched environment in early life can modify caecal microbiota structure and animal response after weaning probably by means of diminishing social stress response.

## Methods

### Animals and study design

This study was performed at an intensive commercial farm, located in Puiggròs, Lleida (Spain). Housing, husbandry and slaughtering conditions conformed to the European Union Guidelines (Directive 2010/63/EU). Experimental procedures were approved by the Animal and Human Experimental Ethical Committee of Universitat Autònoma de Barcelona (UAB; permit code CEEAH 1406) and designed in compliance with the ARRIVE guidelines.

A total of 48 Danbred sows were selected and randomly allotted into two groups with similar distribution of parity times (24 sows per group, 10 primiparous and 14 multiparous). The sows were confined in farrowing crates from 7 days before expected parturition date until weaning. They were distributed across six rooms (3 for multiparous and 3 for primiparous), with ten pens per room and a balanced distribution of treatments by pen. Farrowing was synchronized and cross-fostering was performed within 24 h after parturition in order to standardize the litter size at 13 to 14 piglets. A differential management was carried out between groups, including a control treatment (CTR), with the usual management, and an enriched treatment (ENR) in which two adjacent farrowing pens from the same parity (primiparous or multiparous) were opened to allow piglet socialization 14 days after birth by removing the separation fences. Three different types of enrichment objects^16^ (two hearty chew dog toys, two squid-shaped toys and two natural ropes per pen) were also placed around the farrowing pens in the ENR groups from birth. Sows were fed twice a day with ad libitum commercial feed and water; piglets were provided with creep feed from two weeks of age and ad libitum water. Piglets were weaned on average at 25 days of age and regrouped randomly based on the treatment group and their body weight into 16 pens (40 piglets/pen (ca. 0.20 m^2^/animal); 8 pens per group). Regrouped pens from the ENR treatment had more familiar pen mates (3.9 ± 0.1 familiar pen mates representing 10.3 ± 0.3%) than from the CON (1.7 ± 0.1 familiar pen mates representing 4.7 ± 0.2%). Same management conditions were applied to all piglets after weaning. Weaners were offered ad libitum commercial feed and water.

### Blood and intestinal sampling

Two samplings were performed throughout the study, two days before weaning (-2 d), and three days after weaning (+ 3 d). Fourteen litters (7 litters per treatment) were randomly selected considering a balanced parity within and between treatment groups. From these litters, one medium-weight male piglet per litter was selected for each sampling. The piglets were sedated with an intramuscular injection containing 20 mg/kg of ketamine (Ketamidor) and 2 mg/kg of xylazine (Xilagesic), and humanely euthanized with an overdose of pentobarbital (Euthasol). Blood samples were collected after opening the abdominal cavity directly from the caudal vena cava and serum was obtained by centrifugation during 15 min at 3500 rpm and stored at -80 °C. Jejunum tissue samples (1 cm^2^) were collected from mid-jejunum (1 m after duodenum), washed thoroughly with PBS, and immediately preserved frozen in 1 mL of RNAlater (Deltalab, Rubí, Spain). Caecal content was also collected directly from the cecum and immediately frozen in dry ice. Tissue and caecal samples were kept at -20ºC until further analysis.

For functional studies, ten additional male piglets per experimental group were selected (balanced for parity) and transported to UAB facilities 2 days after weaning. Transport was carried out under sedation by means of xylazine (2.2 mg/kg BW) and Zolazepam-Tiletamine (Zoletil; 8 mg/kg) given intramuscularly. Once in the UAB, piglets were group-housed and offered free access to water and the same commercial feed as were receiving in the farm. One day after (+ 3d), euthanasia was performed by means of an overdose of pentobarbital and fresh colon samples were collected and placed in carbogenated Krebs buffer in order to perform functional studies of the intestine. Each functional experiment was conducted with one animal of each group and sampling order was alternated between groups in each experiment. Although at this age, it is not expected that sex had a relevant impact, sampling and functional studies were only performed in male pigs to minimize residual variability.

### DNA extraction and 16S rRNA gene sequencing

DNA was extracted from 250 mg of each caecal sample using the QIAamp DNA Stool Mini Kit (Qiagen, Hilden, Germany) according to the manufacturer’s instructions following the optimization steps. DNA concentration and purity were checked with NanoDrop ND-1000 spectrophotometer (NanoDrop Technologies, Wilmington, DE, USA). For high-throughput sequencing of caecal microbiota, the MiSeq Reagent Kit V2 (500-cycle) (Illumina, San Diego, CA, USA) was used and the V3-V4 region of 16S rRNA targeted. All subsequent steps were performed on the MiSeq Illumina instrument.

### Sequencing data bioinformatics

The sequence reads generated were processed using Quantitative Insights Into Microbial Ecology (QIIME) version 1.9.1 software. The paired-end reads were merged using join_paired_ends.py using the fastqjoin.py tool. Quality filtering of reads was performed using split_libraries_fastq.py allowing the maximum unacceptable Phred quality score of Q20. The remaining reads were clustered into OTU using UCLUST by subsampling open-reference OTU picking at 97% identity with bacterial 16S GreenGenes (v. 13_8) reference database. The percent of failure sequences to include in the subsample to cluster de novo was set at 0.1. Sequence alignment and phylogenetic tree building were obtained through UCLUST and FastTree. Chimeric sequences were removed via identify_chimeric_seqs.py with ChimeraSlayer as default. Further filtering was performed using filter_otus_from_otu_table.py setting the minimum total OTU observation count at 0.005% as recommended by Bokulich et al^54^.

### RNA extraction and cDNA preparation

Total RNA was obtained from 100 mg of frozen jejunum tissue with the RiboPure kit (Ambion, Foster City, CA, USA) following the manufacturer’s protocol. The subsequent steps of RNA extraction and cDNA preparation procedures were carried out as described previously by Reyes-Camacho^55^.

### Plate design and gene expression study by qPCR

A custom Open-Array plate (Applied Biosystems, Foster City, CA, USA) was designed with a total of 56 selected genes related to intestinal health (Supplementary Table S4). Details regarding genes and primers can be found in previous published work^55^. Multiplex real-time qPCRs were performed in a QuantStudio 12 K Flex Real-Time PCR system (ThermoFisher Scientific, Waltham, MA) using TaqMan Open-Array Real-Time PCR Custom Assays. A final cDNA volume of 6 μl from each sample was transferred to 384-well plates and analysed per duplicate. One sample was used as an inter-plate control to check the replication of results from different plates.

Gene expression data analysis was performed as specified by Reyes-Camacho et al^55^.

### Nuclear magnetic resonance spectroscopy

NMR samples were prepared by mixing 400 μL of serum with 200 μL of a saline buffer 0.9% NaCl (wt/vol) in D_2_O directly in the 5 mm NMR tube^56^. NMR experiments were carried out on a Bruker AVANCE II 600 spectrometer operating at 14.1 T (600.13 MHz frequency for ^1^H), equipped with a z-axis pulsed field gradient 5 mm triple channel probe (TBI), BACS 60 automatic sample changer and a BCU-Xtreme unit for temperature control. The probe temperature was maintained at 300.0 K for all experiments.

Data were collected using the presat PROJECT experiment^57^, a T2-filtered experiment with water signal suppression which attenuates broad signals from high molecular weight. The experiment minimizes J-modulation by using perfect echoes^58^ instead of the standard spin-echoes used in the standard CPMG (Carr-Purcell-Meiboom-Gill) pulse sequence^59,60^. The overall experimental time for each spectrum was 15 min 17 s; acquired using 256 transients with a recovery delay of 2 s and a T2-filter time of 128 ms. Data were collected into 32 K data points and setting a spectral width of 12,019.23 Hz which results in an acquisition time of 1.36 s.

### ^1^H-NMR data pre-processing

Spectra were pre-processed prior statistical analysis using TOPSPIN 3.6 (Bruker BioSpin, Germany). An exponential Fourier Transform using a line broadening factor of 0.3 was used. Lactate signal was used for calibration (1.33 ppm), automatic phase and baseline correction were applied with manual refinement when necessary. Then, spectra were transferred to AMIX 3.9 software where water region from 4.78 to 4.66 ppm was removed, and normalization to total area applied. Finally, a bucket table, containing 250 area regions of 0.04 ppm wide, was extracted to perform statistical analysis on it.

### Ussing chamber experiments

Colon mucosa was stripped from the muscle layers and myenteric plexus, opened along the mesenteric border and divided into 1.5 cm^2^ flat segments, excluding Peyer’s patches. The pieces were mounted in Ussing chambers (World Precision Instruments, Aston, UK) as described by Fernández-Blanco et al.^61^, with minor changes described below. Strips were bilaterally bathed with 5 mL of carbogenated (95% 02 and 5% C02) and warmed (37 ± 1 °C) Krebs buffer. A voltage step of 1 mV was applied every 30 min and the change in I_sc_ was used to calculate tissue conductance (G) and its reciprocal, transepithelial resistance (TEER), by Ohm’s law. Tissues were allowed to stabilize for 30—40 min before baseline values of PD, I_sc_ and G were recorded. Basolateral samples (250 µL, replaced by 250 µL of Krebs buffer) were taken at 30-min intervals during the following 120 min experimental time.

### Statistical analysis

The statistical analysis of caecal microbiota was performed in open-source software R v3.5.3. (R Foundation for Statistical Computing, Vienna, Austria). Support for QIIME in R was achieved through the *phyloseq* package^62^. Alpha diversity analysis was performed using *vegan*^63^ and *microbiome*^64^ packages from raw counts (OTU level), including observed species, Chao1, Shannon and Simpson indices. For beta diversity, measurements were calculated using the Whittaker index^65^ and the *betadisper* function of the vegan package using relative abundances. To compare any differential effects an ANOVA analysis was performed for richness and alpha diversity. A non-metric multidimensional scaling (NMDS), an analysis of similarities (ANOSIM), a permutational analysis of variance (PERMANOVA) and unweighted pair group method with arithmetic mean (UPGMA) hierarchical clustering, all based on Bray–Curtis distance, were also performed for ordination and beta diversity analysis. Cumulative sum scaling (CSS)^66^ normalization of raw counts and differential abundance analysis were performed following the *metagenomeSeq* package pipeline^67^. Taxa were aggregated at phylum, family and genus level and expressed as compositional data. Relative abundances were used to plot taxon abundances. Statistical significance was assumed at *P* < 0.05. The parity number (primiparous/multiparous) was initially included in the different statistical approaches but did not show any significant impact on the data.

For gene expression statistical analysis, RQ values were checked for normalization with R 3.5.3 software and log2 transformation was applied. Two-way ANOVA was performed and Benjamini–Hochberg false discovery rate (FDR) was used to adjust *P* values. Statistical significance was assumed at FDR < 0.05.

Concerning NMR statistical analysis, integral data from bucket table was introduced to SIMCA 14.1 software for multivariate analysis. PCA was applied to the Pareto-scaled data. OPLS-DA was performed to identify potential metabolites differences between pre-defined groups. The validity and the degree of overfit for the OPLS-DA model was made by 100 permutation test and by cross validation. To analyse the performance of classification and discrimination of the OPLS-DA model, a ROC plot was performed. NMR spectra area regions (0.04 ppm) contributing to separation between classes in OPLS-DA model were identified by VIP-plot and S-plot, bucket regions with VIP values ≥ 0.75 and which its spots were located high up or low to the left corner of the S-plot, were chosen.

The integration of gene expression, metagenomics and metabolites was performed by using the open-source software R v3.6.1 and the LinkHD package^68^, which was designed to integrate multiple heterogeneous datasets. For this, three data matrices were prepared with raw OTU counts, gene expression and NMR results. The pipeline established by the program was followed and samples were stratified into clusters. The sample cluster classification derived from the compromise structure was employed to perform the variable selection based on the regression biplot and differential abundance testing.

In Ussing chamber experiments, 2 to 4 colonic strips were studied for each animal and a mean was calculated for each animal. Electrophysiological parameters and FD4 slope were analysed through a t-test (Mann–Whitney test). FD4 kinetics was compared between groups using a two-way ANOVA. Data are expressed as mean ± SEM. Data were considered significant when *P* < 0.05. n values represent different experimental animals. Statistical analysis was performed with GraphPad Prism version 6.01, (GraphPad Software, San Diego, CA, USA).

## Supplementary Information

Supplementary Information

## Data Availability

The datasets generated during and/or analysed during the current study are available from the corresponding author on reasonable request.
