# Supplementary material for: Early socialization and environmental enrichment of lactating piglets affects the caecal microbiota and metabolomic response after weaning
Source: Sci Rep. 2021 Mar 17;11:6113. doi: 10.1038/s41598-021-85460-7 (PMC7969613; doi:10.1038/s41598-021-85460-7)
Supplement: Supplementary file 1 — Supplementary Information [file 41598_2021_85460_MOESM1_ESM.pdf]

Early socialization and environmental enrichment of lactating piglets affects the caecal microbiota and metabolomic response after weaning

Saladrigas-García, M.[1], D'Angelo, M. [1], Ko, H.L. [1], Traserra, S. [2], Nolis, P. [3], Ramayo-Caldas, Y.[4], Folch, J.M. [5,6], Vergara, P. [2], Llonch, P. [1,] Pérez J.F. [1], Martín-Orúe, S.M. [1] *

[1] Animal Nutrition and Welfare Service, Department of Animal and Food Science, Universitat Autònoma de Barcelona (UAB), 08193 Bellaterra, Spain

[2] Department of Cell Biology, Physiology and Immunology, Universitat Autònoma de Barcelona (UAB), 08193 Bellaterra, Spain

[3] Nuclear Magnetic Resonance (SeRMN), Universitat Autònoma de Barcelona (UAB), 08193 Bellaterra, Spain

[4] Animal Breeding and Genetics Program, Institute for Research and Technology in Food and Agriculture (IRTA), Torre Marimon, 08140 Caldes de Montbui, Spain.

[5] Plant and Animal Genomics, Centre for Research in Agricultural Genomics (CRAG), CSIC-IRTA-UAB-UB Consortium, 08193 Bellaterra, Spain.

[6] Department of Animal and Food Science, Universitat Autònoma de Barcelona (UAB), 08193 Bellaterra, Spain.

* Corresponding author (susana.martin@uab.cat)

Supplementary information

Supplementary tables

Supplementary Table S1. Phylum relative counts for suckling and weaned piglets (from highest to lowest considering the global average) and for conventional reared piglets (CTR) or with environmental enrichment (ENR). All the detected phyla are included.

	LACT				WEAN			
	CTR	ENR	SEM	adjPvalues	CTR	ENR	SEM	adjPvalues
Firmicutes	38.18	39.45	3.264	0.9626	57.37	43.31	4.182	0.6236
Bacteroidetes	43.69	42.74	3.080	0.9626	30.04	36.35	2.998	0.5997
Proteobacteria	6.32	6.52	0.889	0.9626	5.18	13.00	2.162	0.4410
Spirochaetes	1.96	4.85	1.064	0.9626	3.83	3.31	0.589	0.9941
Fusobacteria	6.80	2.63	1.953	0.7823	0.03	1.96	0.644	0.3571
Planctomycetes	0.34	0.77	0.201	0.9177	0.48	0.19	0.120	0.9228
Cyanobacteria	0.33	0.97	0.297	0.7823	0.32	0.09	0.082	0.5997
Synergistetes	0.47	0.78	0.193	0.9369	0.13	0.26	0.051	0.6236
Verrucomicrobia	0.68	0.29	0.235	0.9634	0.31	0.33	0.083	0.9990
Actinobacteria	0.26	0.11	0.094	0.9626	0.54	0.25	0.113	0.5997
Tenericutes	0.18	0.10	0.036	0.9177	0.67	0.17	0.180	0.4410
Elusimicrobia	0.44	0.14	0.129	0.9177	0.14	0.27	0.103	0.5997
Lentisphaerae	0.16	0.31	0.084	0.9626	0.01	0.07	0.020	0.4410
Chlamydiae	0.00	0.01	0.005	0.6237	0.45	0.01	0.127	0.1537
Deferribacteres	0.04	0.09	0.024	0.6237	0.07	0.23	0.066	0.5413
Fibrobacteres	0.02	0.09	0.038	0.9177	0.19	0.13	0.051	0.9990
TM7	0.01	0.11	0.041	0.4849	0.15	0.02	0.036	0.4390
Euryarchaeota	0.05	0.03	0.008	0.9626	0.07	0.04	0.015	0.5997
WPS-2	0.07	0.01	0.032	0.9177	0.01	0.01	0.003	0.5413

Supplementary Table S2. Genus relative abundance counts for suckling and weaned piglets (from highest to lowest considering the global average) and in conventional reared piglets (CTR) or with environmental enrichment (ENR). Only predominant genera (+1%) are included.

	LACT				WEAN			
	CTR	ENR	SEM	adjPvalues	CTR	ENR	SEM	adjPvalues
Prevotella	18.78	13.66	2.243	0.9573	11.93	10.06	2.015	0.9432
[Prevotella]	6.72	6.21	1.080	0.9573	5.28	12.57	1.650	0.4413
Bacteroides	5.90	8.12	1.507	0.9573	2.50	2.66	0.542	0.8931
Phascolarctobacterium	3.33	3.30	0.591	0.9842	4.77	3.23	0.430	0.4413
Campylobacter	1.26	1.10	0.422	0.9573	1.45	8.29	2.092	0.3842
Fusobacterium	6.81	2.63	1.954	0.7993	0.03	1.96	0.645	0.3842
Oscillospira	2.65	2.30	0.333	0.9842	2.57	2.80	0.302	0.9432
Treponema	0.81	2.98	0.863	0.9573	3.16	1.46	0.517	0.6459
p-75-a5	0.72	0.47	0.250	0.9573	3.38	2.47	0.868	0.9432
Lactobacillus	2.72	2.07	0.718	0.9573	0.43	0.82	0.325	0.5380
Megasphaera	2.67	1.86	0.685	0.9573	0.40	0.83	0.316	0.8931
Sphaerochaeta	1.15	1.88	0.466	0.9573	0.67	1.86	0.411	0.5266
Roseburia	0.21	0.17	0.076	0.9573	3.05	1.18	0.703	0.5380
Anaerovibrio	0.94	0.38	0.448	0.9573	1.80	0.94	0.286	0.5266
CF231	1.23	1.09	0.249	0.9573	0.60	0.69	0.164	0.8931
Desulfovibrio	0.73	0.68	0.108	0.9573	1.15	0.77	0.259	0.9432
Butyricimonas	0.59	1.14	0.308	0.9573	0.12	0.61	0.153	0.4746
Ruminococcus	0.33	0.30	0.085	0.9573	1.18	0.65	0.193	0.5226

Supplementary Table S3. PCA and OPLS-DA models parameters for ^{1}H-NMR serum profiles of weaned piglets

Enriched vs control P-value	n	PCA			OPLS-DA			
		Number of components	$R^2x_{(cum)}$	$Q^2_{(cum)}$	Number of components	$R^2x_{(cum)}$	$R^2y_{(cum)}$	$Q^2_{(cum)}$
Total spectre	7	1+1	0.64	0.31	1+1	0.62	0.57	0.28
≤ 0.20	7	2	0.85	0.76	1+1	0.83	0.57	0.33

≤ 0.18	7	2	0.88	0.81	1+1	0.87	0.52	0.28
≤0.16	7	2	0.89	0.83	1+1	0.88	0.52	0.29
≤0.14	7	2	0.88	0.81	1+1	0.86	0.61	0.32
≤0.12	7	2	0.90	0.81	1+1	0.86	0.66	0.34
≤0.10	7	2	0.90	0.81	1+1	0.86	0.66	0.34
≤0.08	7	2	0.90	0.81	2	0.89	0.68	0.47
≤0.06	7	3	0.95	0.83	2	0.91	0.68	0.53

Supplementary Table S4. Brief description of the genes analysed.

Gene abbreviation	Gene full name	Functional group
**OCLN**	Occludin	Intestinal barrier
**ZO1**	Zonula occludens 1	Intestinal barrier
**CLDN1**	Claudin-1	Intestinal barrier
**CLDN4**	Claudin-4	Intestinal barrier
**CLDN15**	Claudin-15	Intestinal barrier
**MUC2**	Mucin 2	Intestinal barrier
**MUC13**	Mucin 13	Intestinal barrier
**TFF3**	Trefoil factor 3	Intestinal barrier
**TLR2**	Toll-like receptor 2	Pattern recognition receptors (PRRs)
**TLR4**	Toll-like receptor 4	Pattern recognition receptors (PRRs)
**IL1β**	Interleukin 1 beta	Immune response
**IL6**	Interleukin 6	Immune response
**IL8**	Interleukin 8	Immune response
**IL10**	Interleukin 10	Immune response
**IL17A**	Interleukin 17	Immune response
**IL22**	Interleukin 22	Immune response
**IFN-γ**	Interferon gamma	Immune response
**TNF-α**	Tumor necrosis factor alpha	Immune response
**TGF-β1**	Transforming growth factor beta 1	Immune response
**CCL20**	Chemokine (C-C motif) ligand 20	Immune response
**CXCL2**	Chemokine (C-X-C motif) ligand 2	Immune response
**IFNGR1**	Interferon gamma receptor 1	Immune response
**REG3G**	Regenerating-islet derived protein 3 gamma	Immune response

**PPARGC1α**	Peroxisome proliferative activated receptor gamma, coactivator 1 alpha	Immune response
**FAXDC2**	Fatty acid hydrolase domain containing 2	Immune response
**GBP1**	Guanylate binding protein 1	Immune response
**HSP27**	Heat shock protein 27	Intestinal homeostasis
**HSP70**	Heat shock protein 70	Intestinal homeostasis
**GPX2**	Glutathione peroxidase 2	Digestive enzyme / hormone
**SOD2**	Superoxide dismutase	Digestive enzyme / hormone
**ALPI**	Intestinal alkaline phosphatase	Digestive enzyme / hormone
**SI**	Sucrase-isomaltase	Digestive enzyme / hormone
**DAO1**	Diamine oxidase	Digestive enzyme / hormone
**HNMT**	Histamine N-methyltransferase	Digestive enzyme / hormone
**ANPEP**	Aminopeptidase-N	Digestive enzyme / hormone
**IDO1**	Indoleamine 2,3-dioxygenase	Digestive enzyme / hormone
**GCG**	Glucagon	Digestive enzyme / hormone
**CCK**	Cholecystokinin	Digestive enzyme / hormone
**IGF1R**	Insulin-like growth factor 1 receptor	Digestive enzyme / hormone
**PYY**	Peptide YY	Digestive enzyme / hormone
**SLC5A1**	Solute carrier family 5 (sodium/glucose cotransporter) member 1	Nutrient transport
**SLC16A1**	Monocarboxylate transporter 1	Nutrient transport
**SLC7A8**	Solute carrier family 7 (amino acid transporter light chain, L System) member 8	Nutrient transport
**SLC15A1**	Solute carrier family 15 (oligopeptide transporter) member 1	Nutrient transport
**SLC13A1**	Solute carrier family 13 (sodium/sulfate symporters) member 1	Nutrient transport
**SLC11A2**	Solute carrier family 11 (proton-coupled divalent metal ion transporter) member 2	Nutrient transport
**MT1A**	Metallothionein 1A	Nutrient transport
**SLC30A1**	Solute carrier family 30 (zinc transporter) member 1	Nutrient transport
**SLC39A4**	Solute carrier family 39 (zinc transporter) member 4)	Nutrient transport
**CRHR1**	Corticotropin releasing hormone receptor 1	Stress indicators

**NR3C1**	Glucocorticoid receptor	Stress indicators
**HSD11B1**	Hydroxysteroid (11-beta) dehydrogenase 1	Stress indicators
**ACTB**	β-actin	Housekeeping
**B2M**	β2-microglobulin	Housekeeping
**GAPDH**	Glyceraldehyde-3-phosphate dehydrogenase	Housekeeping
**TBP**	TATA-Box binding protein	Housekeeping

Supplementary figures

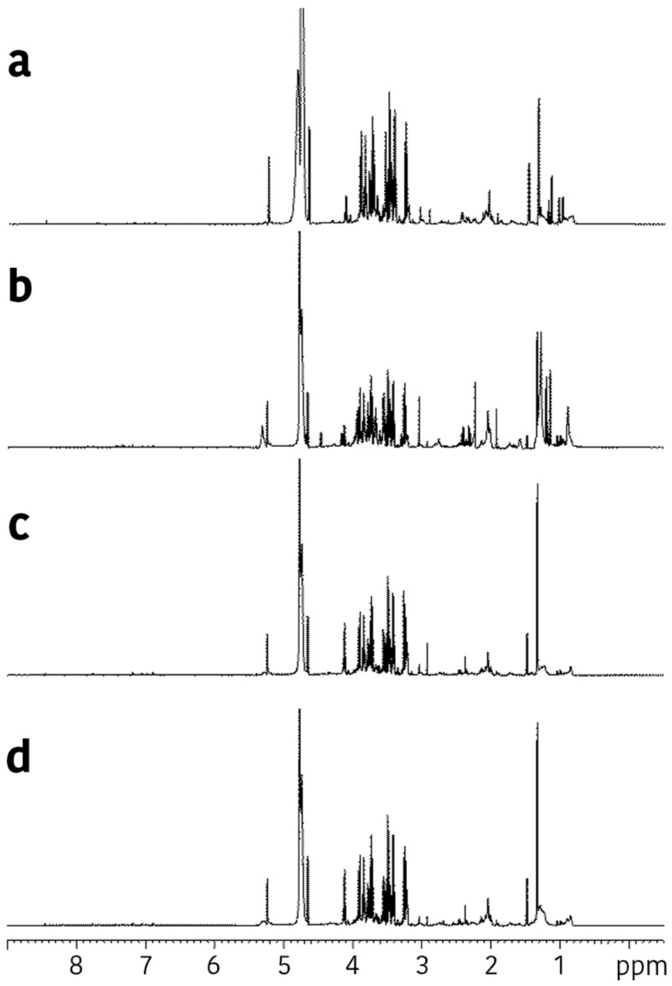

Supplementary Figure S1. Representative ^{1}H-NMR spectra of serum from piglets of different experimental treatment. (**a**) Lactating piglets control group; (**b**) lactating enriched piglets' group; (**c**) weaned piglets control group and (d) weaned enriched piglets' group. All spectra were acquired using a 600 MHz spectrometer.

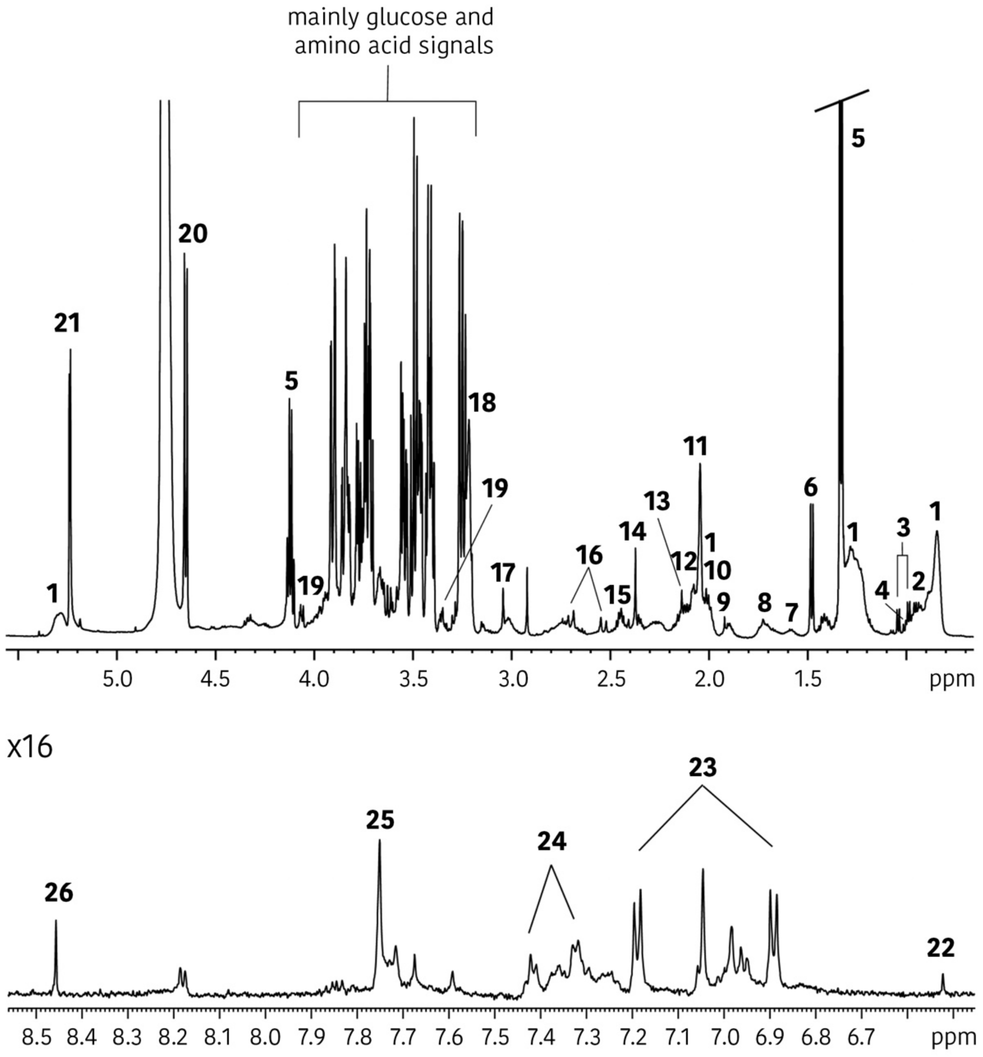

Supplementary Figure S2. Representative ^{1}H CPMG spectrum (600MHz) of serum from nursing piglet. Assignments: **1**, LDL/VLDL; **2**, leucine; **3**, valine; **4**, isoleucine; **5**, lactate; **6**, alanine; **7**, adipate; **8**, arginine; **9**, acetate; **10**, proline; **11**, N-acetyl glycoproteins; **12**, O-acetyl glycoproteins; **13**, glutamine/glutamate; **14**, pyruvate; **15**, glutamate; **16**, citrate; **17**, creatine; **18**, choline; **19**, myo-inositol; **20**, β-glucose (anomeric proton); **21**, α-glucose (anomeric proton); **22**, fumarate; **23**, tyrosine; **24**, phenylalanine; **25**, methyl histidine; **26**, formic acid.

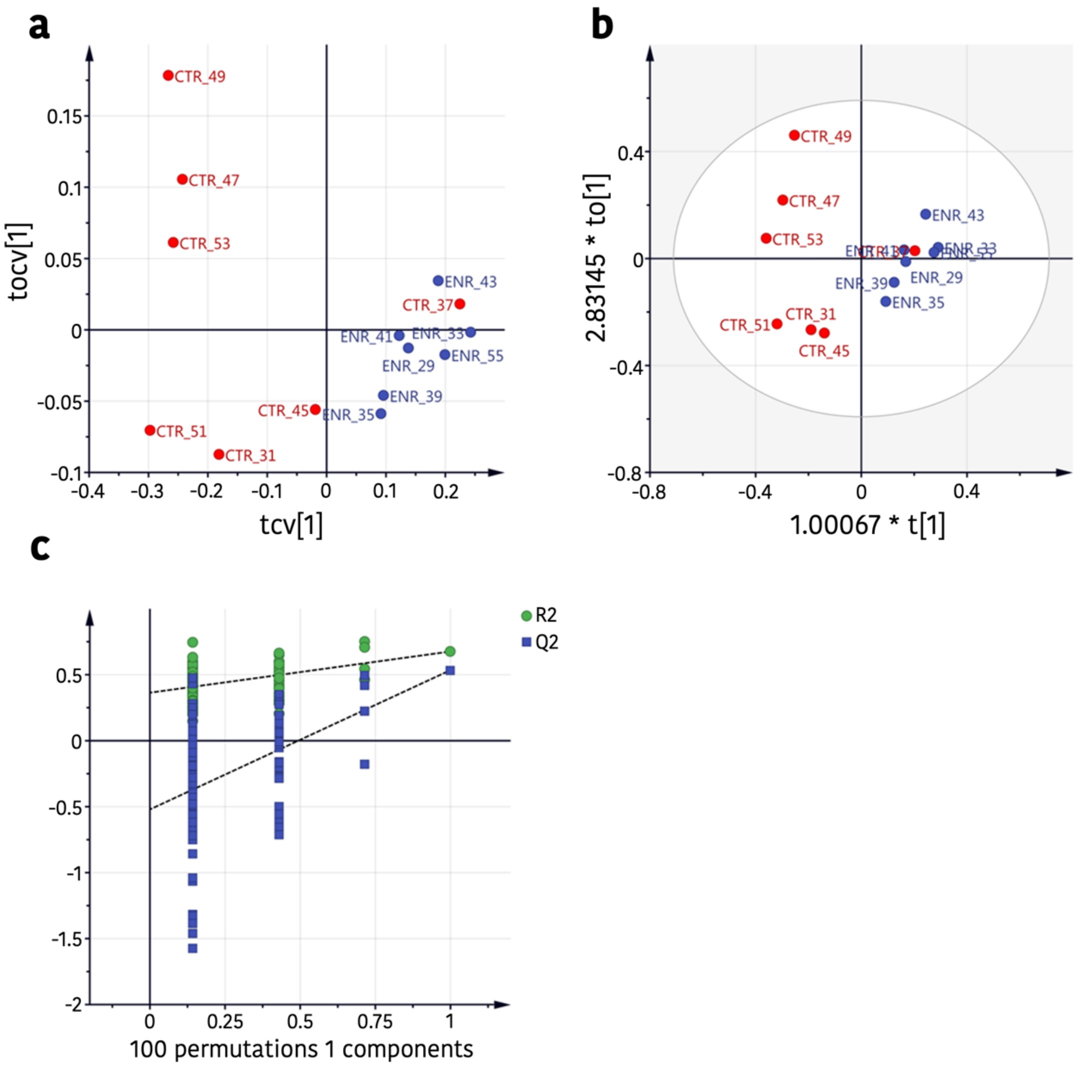

Supplementary Figure S3. Validation of the OPLS-DA model between enriched and control piglets at after weaning period. OPLS-DA (P≤0.06) plot (**a**) derived from ^{1}H-NMR serum spectra of weaned enriched piglets (blue) and weaned control group (red). Cross validation plot (**b**) of the OPLS-DA (P≤0.06) model. 100 random permutation test plot (**c**) relative to OPLS-DA (P≤0.06) model for all samples including enriched and control piglets, where the vertical axis corresponds to R^2 (green circles) and Q^2 (blue squares) values for the model and the horizontal axis corresponds to the correlation coefficient between the original *Y* and the permuted *Y*.

The OPLS-DA (P≤0.06) model constructed to discriminate between enriched and control piglets after weaning was confirmed by cross-validation, the score plot of the regular scores (**Supplementary Fig. S3a**) compared with the score plot of the CDs course (**Supplementary Fig. S3b**) were are almost the same with very little shifting of the spots which is a strong indication that the OPLS-DA (P≤0.06) model is devoid of influential observations and it is very stable to the inclusion or exclusion of all the different observations. Furthermore, the plot of permutation test (100 times) (**Supplementary Fig. S3c**) performed for all samples including enriched piglets and

control piglets shows that the new parameters (R^2=0.36 and Q^2= -0.52) were lower than the original values indicating a lack of over-fitting.

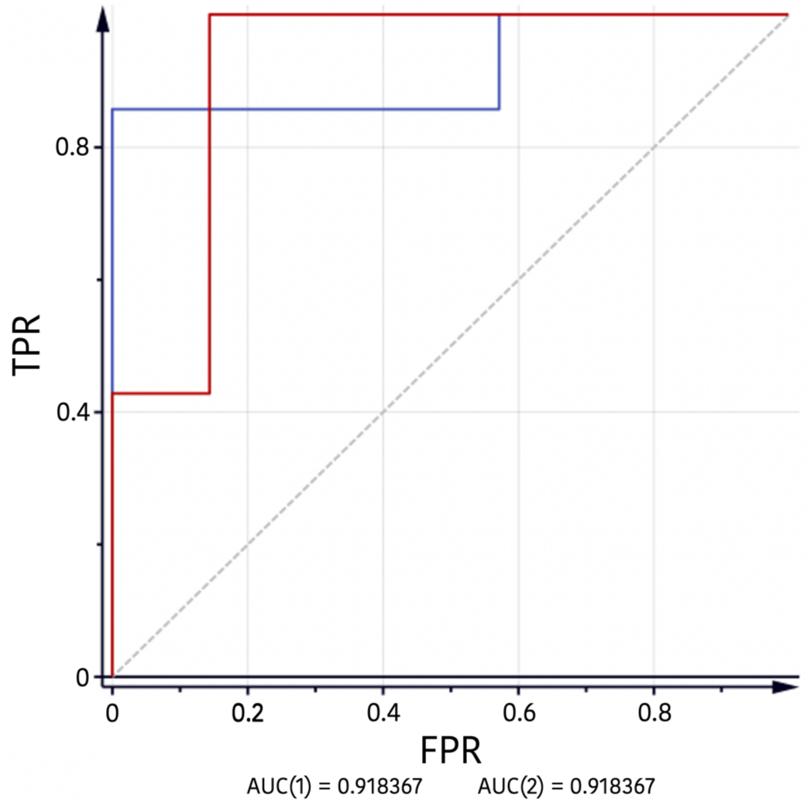

Supplementary Figure S4. Receiver operating characteristic (ROC) plot for the OPLS-DA (P≤0.06) model at after weaning period. Weaned enriched piglets (blue) and weaned control group (red).

The operating characteristic (ROC) plot (**Supplementary Fig. S4**) for the OPLS-DA (P≤0.06) model displays the true positive classification rate (TPR) for enriched group classification (blue) or for control group classification (red) by the constructed model plotted against the corresponding false positive classification rate (FPR) at various threshold settings of the criterion parameter (YPredPS). Thus, for reed curve, TPR (or Sensitivity) represents the probability that a test result will be positive when the enrichment practice is present and TNR (or Specificity) corresponds to the probability that a test result will be negative when the enrichment practice is not present. Every point on the ROC curves represents a pair sensitivity/specificity values corresponding to a particular decision threshold. For both curves the area under the curve (AUC) of the ROC plot has a value of 0.92 indicating high sensitivity and specificity and thus, a high prediction power of the model.

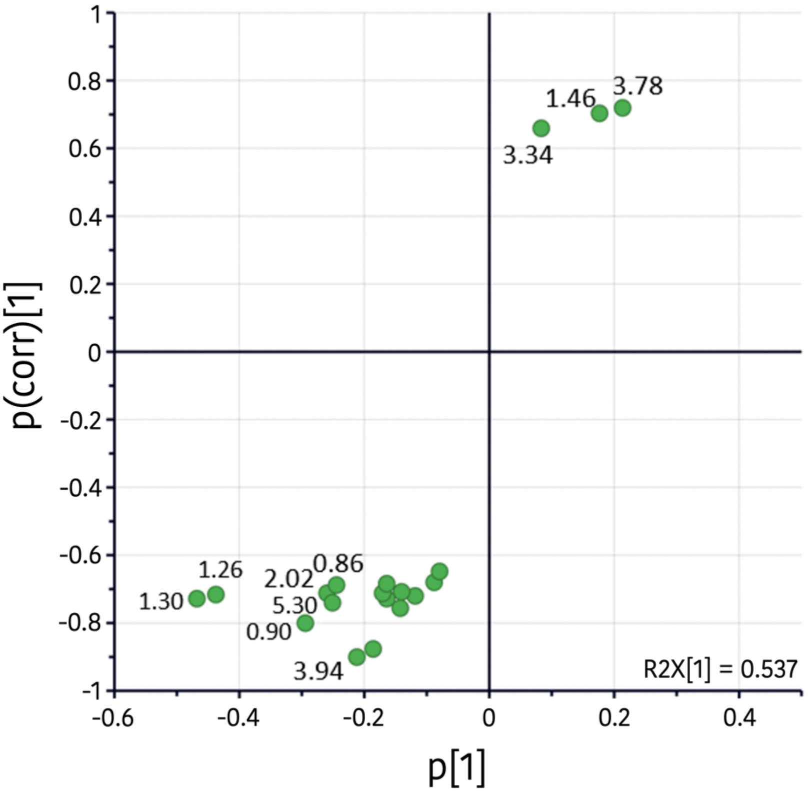

Supplementary Figure S5. S-plot corresponding to OPLS-DA (P≤0.06) model between enriched and control piglets at after weaning period. The covariance value for each variable included is represented on the horizontal axis in the model. The vertical axis represents the correlation values obtained with respect to the dependent variable. The points at the ends of the S-plot curve indicate higher contributions to the classification.
